# The first step into phenolic metabolism in the hornwort *Anthoceros agrestis*: molecular and biochemical characterization of two phenylalanine ammonia-lyase isoforms

**DOI:** 10.1007/s00425-022-03944-w

**Published:** 2022-07-07

**Authors:** Soheil Pezeshki, Ina Warmbier, Tobias Busch, Elke Bauerbach, Peter Szövenyi, Maike Petersen

**Affiliations:** 1 Institut für Pharmazeutische Biologie und Biotechnologie, Philipps-Universität Marburg, Robert-Koch-Str. 4, 35037 Marburg, Germany; 2 Institut für Systematische und Evolutionäre Botanik, Universität Zürich, Zollikerstrasse 107, 8008 Zurich, Switzerland

**Keywords:** *Anthoceros agrestis*, Histidine, Hornworts, Phenylalanine, Phenylpropanoid metabolism, Tyrosine

## Abstract

**Main conclusion:**

Two isoforms of phenylalanine ammonia-lyase (PAL) have been isolated as cDNA sequences from the hornwort *Anthoceros agrestis*. The encoded enzymes convert l-phenylalanine and to lower extents l-tyrosine and l-histidine. Thus, the functional presence of the general phenylpropanoid pathway in one of the earliest land plant groups is established.

**Abstract:**

The hornwort *Anthoceros agrestis* has an elaborated phenolic metabolism resulting in phenolic compounds, such as rosmarinic acid or megacerotonic acid. The general phenylpropanoid pathway is involved in the biosynthesis of these compounds. Two phenylalanine ammonia-lyase (PAL) genes, *AaPAL1* and *AaPAL2*, have been identified in *Anthoceros agrestis* and the protein with an *N*-terminal 6xHis-tag heterologously synthesized in *Escherichia coli* for a full biochemical characterization. Both PAL proteins accept l-phenylalanine, l-tyrosine as well as l-histidine as substrates, although the activity is explicitly the highest with l-phenylalanine. *K*_m_ values as well as catalytic efficiencies were determined for phenylalanine (*K*_m_ AaPAL1 39 µM, AaPAL2 18 µM) and tyrosine (*K*_m_ AaPAL1 3.3 mM, AaPAL2 3.5 mM). In suspension cultures of *Anthoceros agrestis*, PAL genes were transcribed in parallel to rosmarinic acid (RA) accumulation and both showed highest abundance in the early growth phase. In a phylogenetic tree, both AaPAL amino acid sequences grouped within a clade with PAL amino acid sequences of diverse origin ranging from non-vascular to vascular plants, while most PALs from eudicots and monocots were mainly found in two other clades. The similarity of the hornwort PAL amino acid sequences to PAL sequences from vascular plants is more than 80% showing a strong conservation within the land plants. With this characterization of PALs from *Anthoceros agrestis* together with former investigations concerning cinnamic acid 4-hydroxylase and 4-coumaric acid CoA-ligase, the functional presence of the general phenylpropanoid pathway in this hornwort is proven.

**Supplementary Information:**

The online version contains supplementary material available at 10.1007/s00425-022-03944-w.

## Introduction

Phenylalanine ammonia-lyase (PAL, EC 4.3.1.24) is an enzyme in the group of aromatic amino acid lyases together with histidine ammonia-lyases (HAL, EC 4.3.1.3), tyrosine ammonia-lyases (TAL, EC 4.3.1.23) and promiscuous phenylalanine/tyrosine ammonia-lyases (PTAL, EC 4.3.1.25). TAL, however, obviously does not occur in plants and PTALs are mostly restricted to the family Poaceae but also occur in some orders of the dicotyledonous plants and ferns (Barros and Dixon 2020). PAL is responsible for the deamination of l-phenylalanine to its corresponding α-unsaturated acid, *t*-cinnamic acid, and ammonia. PALs are ubiquitous in seed plants, linking primary to secondary metabolism by channeling C6–C3 structures from phenylalanine to coenzyme A-activated 4-coumaric acid (4-coumaroyl-CoA), the well-known general phenylpropanoid metabolism (Hahlbrock and Scheel 1989). The first plant PAL was isolated and characterized from barley (*Hordeum vulgare*) by Koukol and Conn (1961). PAL has also been found in prokaryotic organisms and fungi (Hyun et al. 2011), and it is suggested that PAL in plants has been acquired by horizontal gene transfer, first from bacteria to fungi and then from arbuscular–mycorrhizal fungi to plants (Emiliani et al. 2009; Barros and Dixon 2020). The ability to synthesize phenolic compounds reaches down to Bacillariophyceae, Haptophyta, Cryptophyta, Rhodophyta and Chlorophyta (Niklas et al. 2017). Recent investigations have demonstrated PAL-like sequences from the charophyte *Klebsormidium*, the liverwort *Marchantia polymorpha* and the moss *Physcomitrium patens*, but in most cases the functionality of the encoded enzymes has not been demonstrated (de Vries et al. 2017; Renault et al. 2019). The product of PAL, *t*-cinnamic acid, is the substrate for cinnamic acid 4-hydroxylase (C4H), a cytochrome P450-dependent monooxygenase, forming 4-coumaric acid. 4-Coumaric acid is then activated by 4-coumaric acid coenzyme A ligase (4CL) to 4-coumaroyl-CoA. 4-Coumaroyl-CoA is a precursor for many plant phenolic metabolites, such as phenylpropanoids, flavonoids, catechins, anthocyanins, lignin and lignans. In tracheophytes, 4-coumaroyl-CoA is important for lignification and, therefore, for cell wall reinforcement (Hahlbrock and Scheel 1989). PTALs from grasses additionally produce 4-coumaric acid directly from l-tyrosine and their occurrence was related to the special composition of grass cell walls with higher levels of syringyl-rich lignin and cell wall-bound coumaric acid residues (Maeda 2016; Barros et al. 2016; Barros and Dixon 2020).

In most cases, PALs are homotetrameric soluble enzymes with a conserved structure of three adjacent amino acids (alanine–serine–glycine) which autocatalytically form the so-called MIO (3,5-dihydro-5-methylidene-4H-imidazol-4-one) group (Ritter and Schulz 2004). The MIO group generates electrophilic potential to catalyze the reaction, the mechanism of which is still controversially discussed. The most accepted mechanism is a E1cB elimination reaction in which the MIO group nucleophilically attacks the substrate’s amino group followed by removal of the β proton by a general acid/base catalysis. This creates a carbanion intermediate which leads to the removal of the α-amino group and formation of the double-bond. A Friedel–Crafts-reaction has also been in discussion (Barros and Dixon 2020).

In seed plants, PAL mostly occurs in gene families. Four PAL genes are found in *Arabidopsis thaliana* (Cochrane et al. 2004) and *Nicotiana tabacum* (Reichert et al. 2009) and more than 20 in *Solanum tuberosum* (Joos and Hahlbrock 1992) and *Solanum lycopersicum* (Chang et al. 2008). Genomic data indicate that also bryophytes and other early diverged plant taxa contain more than one gene copy. Gene duplication events have been made responsible for this abundance of PAL genes. Interestingly, only one PAL gene copy has been described in *Pinus taeda* (Whetten and Sederoff 1992). Phylogenetic analyses have shown that PAL genes are grouped into gymnosperm and angiosperm lineages and the angiosperm lineage divided into monocot and eudicot groups (Wu et al. 2014; Dong et al. 2016). Non-spermatophytic plants such as the moss *Physcomitrium patens* and the lycophyte *Selaginella moellendorffi* also have a number of putative PAL sequences which—to our knowledge—have not yet been characterized biochemically, thus the accepted substrates are unknown. Light-controlled PAL activity has been demonstrated in an early physiological study of the moss *Funaria hygrometrica* (Meyer and Angerman 1973). Recently, PAL from the liverwort *Plagiochasma appendiculatum* has been characterized on molecular and biochemical levels (Yu et al. 2014). Six PAL genes from the liverwort *Marchantia polymorpha* were cloned and found to be active in vivo and responding to wounding stress, but no further biochemical characterization has been reported (Yoshikawa et al. 2018). Reports from hornwort PALs are missing to date.

The hornwort *Anthoceros agrestis* from the family Anthocerotaceae (division Anthocerotophyta) is known for its elaborated hydroxycinnamic acid constituents in thalli and suspension cell cultures, such as rosmarinic acid (RA) and its glucoside, megacerotonic acid and anthocerodiazonin (Takeda et al. 1990; Trennheuser et al. 1994; Vogelsang et al. 2006) (Suppl. Fig. S1). *Anthoceros agrestis* forms a small thallus of appr. 7 cm diameter and up to 3 cm height. It consists of a lobed haploid gametophyte and large horn-shaped sporophytes and occurs worldwide on damp and humid fields, usually after harvesting (Bisang 1998). Similar to other members of the division, *Anthoceros* has stomata on the sporophyte and on the ventral side of the gametophyte. A special feature in Anthocerotophyta is the occurrence of pyrenoids, sub-cellular structures only common with green algae, which consist of ribulose-1,5-bisphosphate carboxylase/oxygenase and are used for carbon concentration (Villarreal and Renner 2012). It is also known that *Anthoceros* has cavities for a symbiotic interaction with *Nostoc* species and fungi (Desirò et al. 2013; Szövényi 2016).

The research on *Anthoceros agrestis* has been promoted and accelerated with the publication of its genome sequence (Szövényi et al. 2015; Szövényi 2016; Li et al. 2020). The current view of the evolutionary relationship among the three divisions of bryophytes with respect to the tracheophytes is that the bryophytes, comprising liverworts, mosses and hornworts, are a monophyletic group and a sister group to the tracheophytes; within the bryophytes, the hornworts form a sister group to mosses and liverworts (setaphytes) (Puttick et al. 2018; Li et al. 2020; Zhang et al. 2020). Knowing this, the elucidation of the biosynthetic pathways of the various secondary metabolites, especially those present in non-vascular as well as vascular plants as, for example, phenolic compounds such as RA is of interest.

Suspension cultures of *Anthoceros agrestis* have been shown to produce rather high amounts of RA as well as lower amounts of rosmarinic acid 3′-*O*-β-d-glucoside (Vogelsang et al. 2006). Experiments with crude protein extracts or microsomal preparations from these cells have shown that a number of enzymes known to be involved in RA biosynthesis (Suppl. Fig. S1) in seed plants are also active in *Anthoceros agrestis*, e.g. PAL, hydroxyphenylpyruvate reductase or C4H (Petersen 2003). In the last few years, we have isolated genes encoding C4H, 4CL and tyrosine aminotransferase (TAT) from *Anthoceros agrestis* and characterized the respective enzymes (Wohl and Petersen 2020a, b; Busch and Petersen 2021). We here report the isolation and characterization of two isoforms of PAL from axenic cell suspension cultures of *Anthoceros agrestis* to complete the general phenylpropanoid pathway in this hornwort.

## Materials and methods

### Plant material

Suspension cultures of *Anthoceros agrestis* were established in CB-M medium (CB-medium according to Petersen and Alfermann (1988) without hormones and NZ amines with 1% sucrose) from original cultures provided by Binding and Mordhorst (1991). The cells were transferred to fresh medium every 7 days by adding 5.5 g cells to a 250 ml Erlenmeyer flask containing 50 ml CB-M medium. The suspension cultures were cultivated at 23 ± 2 °C on an orbital shaker at 110 rpm under ambient light conditions.

### Isolation of RNA and synthesis of cDNA

Total RNA was extracted from suspension-cultured cells essentially according to the protocol of Chomczynski and Sacchi (1987). cDNA synthesis was performed with the RevertAid™ First Strand cDNA synthesis kit (ThermoFisher) according to the manufacturer’s protocol.

### Amplification of internal PAL sequences and RACE–PCR

The degenerate primers AaPAL-f1, AaPAL-f2 and AaPAL-r (for primer sequences see Suppl. Table S1; all primers were synthesized by Eurofins Genomics) for the amplification of internal *PAL* sequences were designed from conserved sequence motifs identified in the alignment of 23 known *PAL* sequences (for accession numbers see Suppl. Table S2). Standard PCR reactions contained in a total volume of 25 µl 1 µl cDNA, 5 µl GoTaq buffer (Promega), 3 µl 25 mM MgCl_2_, 0.5 µl 10 mM dNTP mix, 0.5 µl of each primer (100 µM) and 0.1 µl GoTaq Polymerase (Promega). The PCR protocol was as follows: 120 s 94 °C, 60 s 56–62 °C, 90 s 70 °C (1st cycle); 30 s 94 °C, 60 s 56–62 °C, 90 s 70 °C (cycles 2–39); 60 s 94 °C, 60 s 56–62 °C, 10 min 70 °C (last cycle). This PCR approach resulted in an amplicon of 608 bp with high similarity to already known *PAL* sequences and was used to design primers for 3′- and 5′-RACE–PCR (Suppl. Table S1). Further gene-specific primer sequences were deduced from *Anthoceros agrestis* genome sequencing data for partial *PAL* sequences (scaffolds 4301, 15078). RACE–PCR was performed with the SMARTer^®^ RACE 5′/3′-Kit (Clontech) according to the manufacturer’s instructions and resulted in two different 3′- and 5′-sequences each. PCR amplicons were ligated into pDrive (Qiagen) and introduced into *E. coli* EZ to yield plasmids for sequencing (Microsynth Seqlab). The full-length sequences of *AaPAL1* and *AaPAL2* were finally amplified using the Phusion^®^ High-Fidelity DNA Polymerase (New England Biolabs) with the primer pairs AaPAL1-f/AaPAL1-r and AaPAL2-f/AaPAL2-r (for primer sequences see Suppl. Table S1) introducing NdeI restriction sites (underlined) for insertion into the pET15b expression vector (Novagen). Expression plasmids containing the full-length sequences in correct orientation were transferred into *E. coli* SoluBL21 (Amsbio) for protein expression. The full-length open reading frames of *AaPAL1* and *AaPAL2* were deposited in GenBank under the accession numbers MN378319 and MN378320.

### Culture characterization: quantitative real-time PCR and rosmarinic acid content

For culture characterization, a series of flasks of *Anthoceros agrestis* suspension cell cultures was established and cultivated as described above. Cells were harvested by suction filtration immediately after inoculation (day 0) and for each of the following 14 days and stored at − 80 °C. The extraction of total RNA was performed using 50 mg plant material ground in liquid nitrogen essentially according to Chomczynski und Sacchi (1987). Each sample was checked for RNA integrity by agarose gel electrophoresis and the RNA concentrations were determined photometrically. Contaminating DNA was digested with DNase I (Fermentas) using 5 µg extracted RNA with 5 µl 10 × reaction buffer with MgCl_2_, 5 µl DNase I (RNase-free) in a total volume of 50 µl with subsequent incubation at 37 °C for 30 min, followed by phenol–chloroform extraction. After determining the RNA concentration, cDNA synthesis with 0.5 µg RNA and 4 µl 5 × qScript cDNA Supermix (quantabio) in a total volume of 20 µl was performed according to the manufacturer’s protocol and samples were diluted 1:5 with H_2_O and stored at − 80 °C.

Primers (Suppl. Table S1) for quantitative real-time PCR were designed to target fragments of < 200 bp. *AaPAL1* and *AaPAL2* were targeted by a shared primer pair, because all attempts to discriminate between them with various primer pairs had not been successful. *Actin* and *serine threonine protein phosphatase 2a regulatory subunit 2a* (*ST-P 2a*) were chosen as reference genes, since other options (glyceraldehyde 3-phosphate dehydrogenase, elongation factor 1α) did not prove promising in preliminary experiments. To pre-qualify the primer sets, standard PCR products were ligated into pDrive (Qiagen) and introduced into *E. coli* EZ for plasmid preparation and sequencing. To distinguish between *PAL1* and *PAL2* PCR products targeted by the same primer pair, *PAL1* amplicons were cut at an ApaI restriction site missing in *PAL2* PCR products. These were electrophoretically separated, independently processed and sequenced. In addition, real-time PCR with the separated *PAL1* and *PAL2* fragments was performed and the melting curves were recorded (Suppl. Fig. S2).

Each real-time PCR reaction consisted of 5 µl 2 × PerfeCTa SYBR Green Supermix (quantabio), 4 µl cDNA template, 200 nM forward primer and 200 nM reverse primer in a total volume of 10 µl and was performed in a 96 well plate qPCR system (PikoReal 96, Thermo Scientific), using the following program: 95 °C 3 min, followed by 95 °C 15 s, 52 °C 45 s, 68 °C 60 s for 50 cycles. Non-template controls (NTC) supplemented with water were measured in parallel. To test for non-specific by-products, a melting curve (50–95 °C) was recorded for each run. After evaluating the PCR efficiencies (Suppl. Fig. S3) by measurement of fourfold dilution series (up to 1:256), cDNA of each timepoint was diluted 16-fold with H_2_O and was measured simultaneously for the target as well as the two reference genes. The obtained C_q_ values (Suppl. Table S3) were used to calculate the fold-change of the combined *PAL1*/*PAL2* expression according to Pfaffl (2001).

On each of the 15 days of the culture period, 0.5 g fresh weight samples (in triplicate) were collected and freeze-dried. RA extraction and quantification was performed as described below.

### Expression of AaPAL1 and AaPAL2 in *E. coli* SoluBL21 and protein purification

*E. coli* SoluBL21 transformed with the expression plasmid pET15b carrying the full-length sequences of *AaPAL1* or *AaPAL2* were cultivated in LB-medium with 100 µg/ml ampicillin in baffled Erlenmeyer flasks at 37 °C, 220 rpm up to an optical density at 600 nm of 0.4. For induction of protein production 1 mM isopropyl-β-D-thiogalactopyranoside (final concentration) was added and the bacteria further cultivated at 25 °C, 220 rpm overnight. Bacteria were harvested by centrifugation (3000 *g*, 4 °C, 5 min), the supernatant discarded and the pellet frozen at − 80 °C. The cells were resuspended in 4 ml binding buffer (50 mM K_2_HPO_4_/KH_2_PO_4_ pH 8.0, 300 mM NaCl, 10 mM imidazole) for 1 g cells and 1 mg/ml lysozyme added. After incubation on ice for 30 min the cells were disrupted by ultrasonication for three times 30 s with intermittent cooling on ice (intensity 100%, 0.3 cycles). The homogenate was centrifuged at 6000 *g* for 10 min at 4 °C and the supernatant collected for further purification. Up to 10 ml crude protein extract were added to 1 ml pre-equilibrated Ni–NTA resin (Novagen) and incubated on ice for 1 h. The resin was rinsed with 16 ml wash buffer 1 (50 mM K_2_HPO_4_/KH_2_PO_4_ pH 8.0, 300 mM NaCl, 20 mM imidazole) and 2 ml wash buffer 2 (50 mM K_2_HPO_4_/KH_2_PO_4_ pH 8.0, 300 mM NaCl, 50 mM imidazole). The bound protein was eluted with 3 ml elution buffer (50 mM K_2_HPO_4_/KH_2_PO_4_ pH 8.0, 300 mM NaCl, 250 mM imidazole). The elution fraction was transferred into boric acid/sodium tetraborate buffer pH 8.8 (78 mM H_3_BO_3_, 30 mM Na_2_B_4_O_7_, 20 mM NaCl) using PD-10 columns (GE Healthcare). Protein concentrations were determined according to Bradford (1976). The purity of the His-tagged protein was monitored by SDS–PAGE essentially according to Laemmli (1970). Gels were stained with Coomassie Brilliant Blue R250 or subjected to Western blotting basically as described by Mahmood and Yang (2012), but using the Towbin et al. (1979) buffer system (Suppl. Figs. S4 and S5). AaPAL1 and AaPAL2 were detected with mouse anti-6xHis-tag monoclonal antibodies (ThermoFisher, MA1-21315) and goat anti-mouse secondary antibodies conjugated to alkaline phosphatase (Life Technologies, A16087). Standard protocols (https://www.sysy.com/protocols/westernblot-ap-detection) were followed for nitro blue tetrazolium chloride/5-bromo-4-chloro-3-indolyl-phosphate staining. The presence of His-tagged protein is indicated by purple staining (Sambrook and Russell 2001).

### Determination of catalytic activities of AaPAL1 and AaPAL2

The catalytic activity of heterologously expressed PAL proteins was tested by measuring the formation of *t*-cinnamic acid from l-phenylalanine (Phe), of 4-coumaric acid from l-tyrosine (Tyr) or of urocanic acid from l-histidine (His) by HPLC or photometrically (for comparison of the three amino acids Phe, Tyr and His). Standard assays for HPLC analysis contained in a total volume of 250 µl boric acid/sodium tetraborate buffer pH 8.8 10 mM Phe and up to 62 µg protein. Assays were incubated at 36 °C for 10 min. The reaction was stopped by addition of 50 µl 6 N HCl and double extraction with 500 µl ethyl acetate each. The combined ethyl acetate phases were evaporated to dryness and redissolved in 100 µl 60% aqueous methanol with 0.01% H_3_PO_4_ for HPLC analysis.

Determination of the pH-optima was performed in 0.1 M K_2_HPO_4_/KH_2_PO_4_ buffers pH 4.5–9.0. For the determination of *K*_m_-values for Phe, the protein concentration was reduced to appr. 0.5/1 µg per assay (PAL2/PAL1) and the concentration of Phe was varied between 1 and 500 µM. Incubation time was kept at 4 min for PAL2 and 10 min for PAL1. For kinetic assays with Tyr, the concentrations were varied between 0 and 9.6 mM (the highest possible concentration under assay conditions due to the restricted solubility). The assays contained appr. 0.5/1.9 µg purified protein (PAL2/PAL1) and were incubated for 15/10 min at 36 °C. Substrate saturation curves were analyzed with the GraphPad Prism 9 software using the Michaelis–Menten and Hanes–Woolf models.

For the determination of the catalytic activity with His in comparison to Phe and Tyr, spectrophotometric assays were performed. The assays contained in 1 ml boric acid/sodium tetraborate buffer pH 8.8 1 µg (Phe) or 5 µg (Tyr, His) PAL1 or PAL2 protein and 5 mM of the respective amino acid and were incubated at 36 °C for 30 min; assays with His additionally contained 7 mM reduced glutathione (Brand and Harper 1976). The absorption was recorded at 290/309/277 nm for Phe/Tyr/His and the activity calculated using extinction coefficients determined for *t*-cinnamic acid (*ε*_290 nm_ = 9.08 cm^2^ mmol^−1^), 4-coumaric acid (*ε*_309 nm_ = 11.44 cm^2^ mmol^−1^) and *t*-urocanic acid (*ε*_277 nm_ = 18.12 cm^2^ mmol^−1^) in the applied buffer system.

### HPLC analysis

HPLC analysis was performed on an Equisil ODS column (length 250 mm, Ø 4 mm with a 20 mm pre-column, particle size 5 µm; Dr. Maisch GmbH, Ammerbuch, Germany) by isocratic elution with 60% aqueous methanol acidified with 0.01% H_3_PO_4_ at 1 ml/min. Eluting compounds were detected spectrophotometrically at 290 nm (*t*-cinnamic acid) or 309 nm (4-coumaric acid). The reaction products were quantified with the help of authentic 25 µM standards. HPLC chromatograms showing PAL assays with l-phenylalanine incubated for 0, 10 and 20 min as well as a *t*-cinnamic acid standard are depicted in Suppl. Fig. S6.

### Construction of a phylogenetic tree

A phylogenetic tree was constructed from (putative) PAL amino acid sequences (see Suppl. Table S4) using the MEGA7 software package (version 7.0.18) and the maximum likelihood method with 1000 bootstraps (Kumar et al. 2016).

### Feeding of l-phenylalanine and l-tyrosine to suspension cultures of *Anthoceros agrestis*

To evaluate the impact of precursor addition to suspension cultures of *Anthoceros agrestis*, Phe (as precursor for the caffeic acid part of RA) or Tyr (as precursor for the 3.4-dihydroxyphenyllactic acid part) or both were fed to the suspension cultures. 2.5 g 7-day-old suspension cells were transferred to 25 ml CB-M medium containing either 1 mM Phe or 1 mM Tyr or both in triplicate. Control cultures were inoculated into normal CB-M medium. Cells were harvested by suction filtration after eight culture days (cultivation at 23 ± 2 °C under shaking at 110 rpm), frozen and lyophilized. The freeze-dried cells were ground to a fine powder by mortar and pestle and duplicate samples of 20 mg extracted with 2 ml 70% ethanol each in an ultrasonic bath at 70 °C for twice 10 min. The samples were centrifuged for 10 min at 16.000 *g* and the supernatant collected. The samples were diluted 1:50 with the HPLC solvent and analyzed as follows: Equisil ODS column 25 cm × 4 mm with pre-column 2 cm × 4 mm; 50% aqueous methanol with 0.01% H_3_PO_4_; flow 1 ml/min; UV detection at 333 nm. RA (25 µM) was used as an authentic standard for identification and quantification.

## Results

### Identification and analysis of PAL sequences from *Anthoceros agrestis*

Using degenerate primers deduced from alignments of *PAL* sequences from different organisms and cDNA synthesized from total RNA isolated from suspension cells of *Anthoceros agrestis* a partial sequence of 608 bp was identified. This sequence was used to construct primers for 5′- and 3′-RACE–PCR resulting in the amplification of two full-length isoforms of *PAL* with 2259 and 2262 bp length. These sequences display an identity to each other of 76.4% on nucleotide level. The encoded proteins have molecular masses of 81,895 Da (AaPAL1; 752 amino acid residues, pI 5.72; including 6 × His-tag: 84,058 Da) and 81,704 Da (AaPAL2; 753 amino acid residues, pI 5.93; including 6 × His-tag: 83,867 Da) (GenBank MN378319 and MN378320) and display an identity to each other of 82.6% and a similarity of 89%. Both sequences show the catalytic triade consisting of the amino acid residues alanine, serine and glycine forming the MIO group which is essential for catalysis (Langer et al. 2001) (Suppl. Fig. S7). The hornwort sequences of AaPAL are both 63% identical and 75% similar to the PAL amino acid sequence of *Melissa officinalis* (Lamiaceae) (Weitzel and Petersen 2010) with the region of highest differences in the *N*-terminal region of the proteins (Suppl. Fig. S7).

Comparison of the identified *PAL* sequences with the genome of *Anthoceros agrestis* (Oxford; https://www.hornworts.uzh.ch/en/hornwort-genomes.html) showed the presence of these sequences which we had identified beforehand as well as a third, very short sequence with appr. 50% similarity to *PAL*. Comparison of the genome sequences of *AaPAL1* (utg000011l) and *AaPAL2* (utg000003l) with our cDNA sequences showed that both *PAL*s each contain two introns. The first intron in *AaPAL1* starts after 445 nucleotides (nt) and thus is a phase 1 intron, it has a length of 173 nt, the second intron is a phase 0 intron after 1236 nt with a length of 140 nt. Intron 1 of *AaPAL 2* is a phase 0 intron starting after 447 nt with a length of 97 nt. The second intron of *AaPAL2* starts after 1164 nt (phase 0) and comprises 118 nt.

In order to demonstrate the phylogenetic relationship of PAL sequences across the plant kingdom, amino acid sequences from PALs with known or supposed catalytic activity were selected and a phylogenetic tree constructed by the maximum likelihood method of the MEGA7 software (Suppl. Table S4 and Suppl. Fig. S8). Besides the outlying sequence of the charophyte *Klebsormidium nitens* roughly three clades were resolved, the first of which contains PALs from eudicotyledonous and monocotyledonous plants as well as one sequence from *Asarum sieboldii* from the magnoliids. From the eudicots, only the PAL1 sequence of *Nelumbo nucifera* groups with the second clade. This PAL1 has already been described as an ancient sequence more similar to gymnosperm PALs (Wu et al. 2014). In the gymnosperms a gymnosperm-specific PAL lineage has evolved according to investigations by Bagal et al. (2012). The third clade only contains PALs from monocots. Most interesting is the second clade which includes PAL sequences from a variety of non-vascular and vascular plants, among them monocotyledonous plants, gymnosperms, gnetophytes, polypodiophytes, lycophytes and bryophytes. The bryophytes comprise liverworts (*Marchantia polymorpha*, *Plagiochasma appendiculatum*) as well as mosses (*Physcomitrium patens*) and also the two PAL sequences from the hornwort *Anthoceros agrestis* described here. Within the bryophytes, the PAL sequences from hornworts and mosses are more closely related within one sub-clade and the liverwort sequences in the sister clade.

### Culture characterization: PAL transcript abundance and RA content

**Fig. 1:**
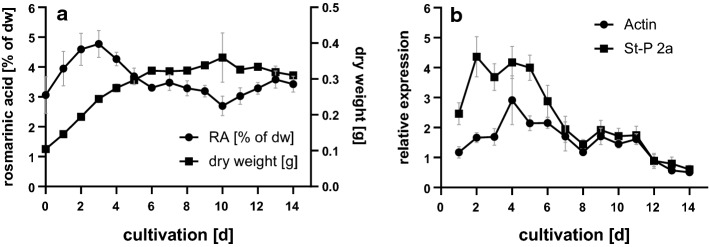
Characterization of an *Anthoceros agrestis* suspension culture during 14 days. **a** Rosmarinic acid content (●; *n* = 6 ± SD) and dry weight (dw) (▲; *n* = 3 ± SD). **b** Combined quantitative real-time PCR of *AaPAL1/2* normalized with the two references genes *actin* (●) and *St-P 2a* (■) according to Pfaffl (2001) (*n* = 4 ± SD)

The *Anthoceros agrestis* cell culture exhibited a growth phase lasting over 14 days with an increase in dry weight until day 10 followed by a stationary phase. This was accompanied by an increase in the RA content from 3% of the dry weight to 4.8% in the first 3 days. Thereafter, it decreased steadily to about 3% of the cell dry weight (Fig. 1a). Combined *PAL1* and *PAL2* expression was analyzed by quantitative real-time PCR (Fig. 1b) since discrimination between the two *PAL* isoforms by PCR was not successful in several attempts. Two reference genes were used for normalization, *actin* and *St-P 2a*, with lower efficiency for *actin* (Suppl. Fig. S3) and less overall stability of C_q_ values in terms of standard deviation. Both plots show an increase in *PAL* expression in the first days of growth. *PAL* shows highest expression between days 3–6 (normalized against *actin*) or 2–5 (normalized against *St-P 2a*) (Fig. 1b) with approximately fourfold expression compared to day 0. During the later cultivation period, the expression decreases with an intermediate second, but lower plateau between days 9 and 11. The melting curve (Suppl. Fig. S2) revealed that two products were formed, these were separated and sequenced and could be clearly identified based on GC content (*PAL1* fragment with 59.0% and *PAL2* fragment with 56.5%) and the resulting deviation in melting temperature.

### Characterization of heterologously expressed AaPAL1 and AaPAL2

Full-length sequences of *AaPAL1* and *AaPAL2* in the expression vector pET15b in *E. coli* SoluBL21 were expressed after induction overnight at 25 °C in order to minimize the formation of inclusion bodies. After disruption of the bacteria, the proteins were purified by metal chelate chromatography. Western blot analysis with antibodies directed against the *N*-terminally attached 6xHis-tag showed proteins around the expected molecular masses (Suppl. Figs. S4 and S5). These highly enriched PAL preparations were used to determine the kinetic characteristics of the two PAL enzymes from *Anthoceros agrestis*. Both AaPALs displayed considerably higher affinities and activities with l-phenylalanine as substrate qualifying them as real PALs. The enzyme assays showed a higher specific activity of AaPAL1 compared to AaPAL2 with Phe as substrate, while AaPAL2 had higher specific activity with the substrate Tyr. The optimal reaction temperature for both PALs was at 60 °C. Due to the relatively fast loss of activity at this temperature, reaction assays were always performed at 36 °C. Similar high temperature optima have been reported for PALs from vascular plants (e.g. Rösler et al. 1997; Weitzel and Petersen 2010). The pH-optimum for both enzymes was determined in 0.1 M potassium phosphate buffer. AaPAL1 displayed a pH-optimum of pH 7.5. The optimum for AaPAL2 was slightly higher at pH 8.0. This is in the range of reported pH-optima for PAL from different plant origins (e.g. Rösler et al. 1997; Cochrane et al. 2004; Weitzel and Petersen 2010).

**Fig. 2:**
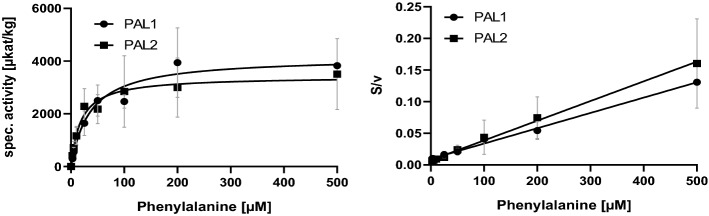
Determination of *K*_m_ and *V*_max_ of AaPAL1 (*n* = 4 ± SD) and AaPAL2 (*n* = 3 ± SD) with l-phenylalanine as substrate determined by Michaelis–Menten (left) and Hanes–Woolf (right) plots; S = substrate concentration, v = reaction velocity

**Fig. 3:**
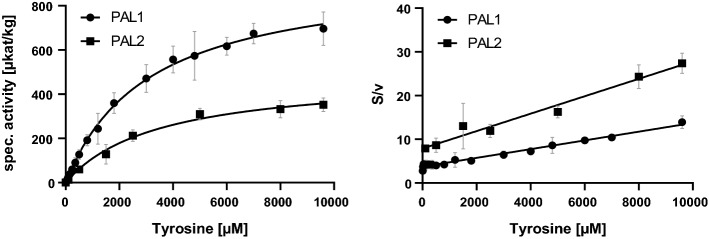
Determination of *K*_m_ and *V*_max_ of AaPAL1 (*n* = 4 ± SD) and AaPAL2 (*n* = 4 ± SD) with l-tyrosine as substrate determined by Michaelis–Menten (left) and Hanes–Woolf (right) plots; S = substrate concentration, v = reaction velocity

Heterologously synthesized AaPAL1 and AaPAL2 proteins were used with the attached His-tag for substrate saturation kinetics. Kinetic parameters were determined from three or four independent measurements and deduced from Michaelis–Menten and Hanes–Woolf plots (Table 1). The *K*_m_ value for Phe was at 39 ± 15 µM for AaPAL1 and 18 ± 4 µM for AaPAL2 showing the higher affinity for Phe of AaPAL2. On the other hand, the *K*_m_ values for Tyr were roughly in the same range for both amino acids and about 100 times higher than for Phe. For tyrosine, the *V*_max_ values differed considerably with AaPAL2 displaying only about half the activity compared to AaPAL1. The catalytic efficiency (*k*_cat_/*K*_m_) was higher for AaPAL2 using Phe as substrate and for AaPAL 1 with Tyr (Table 1 and Figs. 2 and 3).

**Table 1:** Kinetic data of AaPAL1 and AaPAL2 using the substrates l-phenylalanine and l-tyrosine (determined from three to four independent experiments) and analyzed with the GraphPad Prism 9 software using the Michaelis–Menten (MM) and Hanes–Woolf (HW) models

Enzyme	Substrate		*K*_m_ [µM]	*V*_max_ [µkat/kg]	*k*_cat_ [1/s]	*k*_cat_/*K*_m_ [1/µM s]
PAL1	l-Phe	MM	39 ± 15	4202 ± 625	0.353	9.05·10^–3^
		HW	39 ± 10	4134 ± 149	0.347	8.90·10^–3^
PAL2		MM	18 ± 4	3430 ± 1293	0.288	16.01·10^–3^
		HW	25 ± 13	3610 ± 1372	0.303	12.13·10^–3^
PAL1	l-Tyr	MM	3308 ± 987	966 ± 85	0.081	2.45·10^–5^
		HW	3844 ± 982	1012 ± 85	0.085	2.21·10^–5^
PAL2		MM	3526 ± 617	494 ± 47	0.041	1.16·10^–5^
		HW	3994 ± 830	506 ± 56	0.042	1.05·10^–5^

The corresponding graphs are shown in Figs. 2 and 3

Since PALs from early diverged plants might be closer to their putative ancestor sequence coding for histidine ammonia-lyase (HAL), we also determined the activities of AaPAL1 and AaPAL2 with His in comparison to Phe. However, His was a very poor substrate. The activities with His were only at 0.4% and 0.2% of the activities with Phe for AaPAL1 and AaPAL2, respectively. Thus, a closer relation to the proposed ancestor protein HAL cannot be shown. HALs from plants have only rarely been described, e.g. from the green algae *Chlamydomonas reinhardtii* and *Dunaliella tertiolecta* (Hellio and Legal 1999; Hellio et al. 2004) as well as a cofactor-dependent HAL from the seed plant *Vicia faba* (Kamel and Maksoud 1978).

### Precursor feeding to suspension cultures of *Anthoceros agrestis*

As Phe and Tyr are reported as the precursors for the biosynthesis of RA, feeding of these amino acids might affect the formation of RA. This might give information about first, whether the concentrations of these amino acids are rate-limiting for RA biosynthesis and second, whether the entry-point enzymes PAL or TAT might catalyze rate-limiting steps. In this feeding experiment, Phe and Tyr or both were fed to the suspension cells for 7 days at concentrations of 1 mM. Analysis of the RA content did not show significant differences between unfed controls and fed cultures (Suppl. Fig. S9). The RA content ranged between 3.35% and 3.70% of the cell dry weight. In a similar experiment performed independently, the overall RA content was higher but again no significant differences were observed.

## Discussion

Phenolic compounds play an important role in the plants’ conquest of land. Thus, investigations on phenylalanine ammonia-lyases (PAL)—although one of the best investigated enzymes of plant specialized metabolism—in early diverged plants is of importance, especially regarding their substrate preferences. PALs are supposed to have evolved from histidine ammonia-lyases and then transferred to plants by horizontal gene transfer, first from bacteria to fungi and then further from arbuscular–mycorrhizal fungi to plants (Emiliani et al. 2009; Barros and Dixon 2020). PAL-like sequences are found in streptophytes (*Klebsormidium nitens*; de Vries et al. 2017) and charophytes (e.g. *Chara braunii*; de Vries et al. 2021) and are sometimes assigned as HALs instead of PALs. Here, the substrate acceptance has to be tested to show the discrimination of accepted substrates between His (the substrate of the ancient predecessor) and Phe. The two PALs from the hornwort *Anthoceros agrestis* described here clearly prefer Phe and only very low residual activity (< 1% of the activity with Phe) is found with His. A comparison to the ability of seed plant PALs to accept His is not easy since this has rarely been tested; some PALs from flowering plants probably have no activity with His at all (own unpublished results).

Phylogenetic analyses of PAL amino acid sequences across the plant kingdom (Suppl. Fig. S8) showed a grouping within a clade mainly containing gymnosperms and seedless vascular plants, whereas PALs from eudicotyledonous plants mainly group in a separate clade. Interestingly, however, the similarity of PAL proteins from *Melissa officinalis* and *Anthoceros agrestis* is high at 75%. The *Anthoceros* PALs were placed in closer neighborhood to *Physcomitrium patens* PALs than to liverworts PAL. PALs from the fern *Psilotum nudum* (Psilotaceae), the clubmoss *Diphasiastrum tristachyum* and the spermatophyte *Cunninghamia lanceolata* (Cupressaceae) are other members of this branch. This picture is not in accordance with the hypothesis that mosses and liverworts are more closely related to each other and hornworts are regarded as earlier diverged sister clade (Puttick et al. 2018; Li et al. 2020; Zhang et al. 2020), but may be due to the small data set used here.

Feeding of the primary precursors Phe and Tyr or both together (at concentrations of 1 mM) showed no relevant effect on the content of RA or onto the growth behavior of suspension-cultured cells of *Anthoceros agrestis* which is able to accumulate high concentrations of RA as well as RA-3-*O*-β-glucoside (Vogelsang et al. 2006). Thus, amino acid concentrations might not be limiting inside the cells. In contrast, in a suspension culture of *Fragaria ananassa*, permanent feeding of Phe at low concentrations had a strong positive effect on the accumulation of anthocyanins (Edahiro et al. 2005). Feeding of Phe or Tyr also slightly increased the content of phenylethanoid glycosides in cell cultures of *Cistanche deserticola* (Hu et al. 2014). The RA accumulation in cell cultures of *Origanum vulgare* was positively influenced by Phe feeding resulting in an increase of RA by 80% under feeding with 0.1 mM Phe (Li et al. 2021). This shows that RA content can in certain cases be increased, which was, however, not the case for *Anthoceros agrestis*.

In this suspension culture of *Anthoceros agrestis* RA accumulation came to its maximum (4.8%) already early in the culture period, on day 3, and then decreased steadily to about 3% of the dry weight (Fig. 1). Earlier descriptions of RA accumulation with a different *Anthoceros agrestis* suspension culture line showed the maximum RA accumulation later, on day 8 of the cultivation period (Vogelsang et al. 2006); here, however, also a different medium (CB2) had been used. Analysis of transcript abundance of *AaPAL*s during the 14-day cultivation period revealed a roughly parallel increase of RA content and transcripts. It must, however, be taken into account that PAL activity is not only needed for the formation of RA. The expression was increased to threefold (using *actin* as reference gene) or 4.5-fold (with *St-P 2a* as reference gene) with comparable courses of the curves (Fig. 1). Although several different primer pairs were used for separate amplification of *AaPAL1* and *AaPAL2*, a total discrimination was not possible. For this reason, we decided to use a primer pair matching to both *PAL*s. Due to the differing GC-content of the amplicons and based on sequencing results we showed, that both PAL isoforms are amplified to a measurable extent.

Biochemical characterization of AaPAL1 and AaPAL2 showed substrate acceptance and substrate affinities in the range of other reported PALs. AaPAL2 showed a higher affinity towards Phe (*K*_m_ 18 µM) and a higher catalytic efficiency (*k*_cat_/*K*_m_ 16 µM^−1^ s^−1^) than AaPAL1 (*K*_m_ 39 µM, *k*_cat_/*K*_m_ 9 µM^−1^ s^−1^). The affinity for Tyr of both AaPALs was about 100 times lower at 3–4 mM and the same is found for the catalytic efficiencies. PAL from the liverwort *Plagiochasma appendiculatum* had a *K*_m_ value of 98 µM for Phe and 5 mM for Tyr; here the acceptance of tyrosine was only 4% of the activity with Phe (Yu et al. 2014). The BRENDA enzyme database (www.brenda-enzymes.org) lists *K*_m_ values between 6.5 µM (*Glycine max*) and 2.6 mM (*Arabidopsis thaliana*) for Phe for PAL from different plant sources. Therefore, AaPALs qualify for typical plant PALs comparable with PALs from spermatophytes.

## Conclusions

Although *Anthoceros agrestis* belongs to the extant representatives of earliest land plants, it has an elaborated phenolic metabolism which is based on the same enzymological toolkit as in seed plants, the general phenylpropanoid metabolism. The work presented here shows that—as described before for C4H and 4CL (Wohl and Petersen 2020a, b)—PAL as an entry enzyme of the core general phenylpropanoid pathway is rather similar to PALs from seed plants. This is reflected in the biochemical properties of both AaPALs. The affinities for the main substrate Phe are in the range observed for several seed plant PALs. A certain although strongly reduced catalytic activity is observed with Tyr and even a very low activity with His. Overall, the properties of *Anthoceros agrestis* PALs show high similarities to seed plant PALs on biochemical and molecular levels. This—together with our earlier results for C4H and 4CL (Wohl and Petersen 2020a, b)—might indicate that the general phenylpropanoid metabolism has been established rather early in land plant evolution and has been conserved. Further investigations on this topic should be performed with green algae of the Charophyceae and Zygnematophyceae as the latest predecessors of land plants.

### Author contribution statement

SP, IW, TB, EB and MP performed experiments and wrote the article. PS provided sequence information after having established the *Anthoceros agrestis* genome sequence database. MP finalized the data and the text. All authors read and approved the manuscript.

## Supplementary Information

Below is the link to the electronic supplementary material.

Supplementary file1 (PDF 535 KB)

## Data Availability

Sequence data were deposited in GenBank (https://https.ncbi.nlm.nih.gov/genbank/) under the accession numbers MN378319 and MN378320. The datasets generated and/or analyzed during the current study are available from the corresponding author on reasonable request.

## Abbreviations

4CL: 4-Coumaric acid coenzyme A ligase
C4H: Cinnamic acid 4-hydroxylase
PAL: Phenylalanine ammonia-lyase
PTAL: Phenylalanine/tyrosine ammonia-lyase
RA: Rosmarinic acid
TAL: Tyrosine ammonia-lyase
