# Supplementary material for: The first step into phenolic metabolism in the hornwort Anthoceros agrestis: molecular and biochemical characterization of two phenylalanine ammonia-lyase isoforms
Source: Planta. 2022 Jul 7;256(2):33. doi: 10.1007/s00425-022-03944-w (PMC9262799; doi:10.1007/s00425-022-03944-w)
Supplement: Supplementary file 1 — Supplementary file1 (PDF 535 KB) [file 425_2022_3944_MOESM1_ESM.pdf]

Supplementary material for

The first step into phenolic metabolism in the hornwort *Anthoceros agrestis*: molecular and biochemical characterization of two phenylalanine ammonia-lyase isoforms

Soheil Pezeshki[1], Ina Warmbier[1], Tobias Busch[1], Elke Bauerbach[1], Peter Szövenyi[2], Maike Petersen[1]

Suppl. Table S1 Sequences of PCR primers used for amplification of *PAL* sequences from *Anthoceros agrestis* and qPCR analysis. *Actin* and *ST-P 2a* primers were deduced on the basis of the following scaffolds: *Actin* AagrOXF_evm.model.utg000050l.84.5, *ST-P 2a* AagrOXF_evm.model.utg000126l.165.1 on https://www.hornworts.uzh.ch

**Primer name**	**Sequence**	**Description**
AaPAL-f1	GARCTCATYCGBTTCCTBAAYGCNGG	Degenerate primers for amplification of internal PAL sequences
AaPAL-f2	GCVHTVTTCTGYGATGTGATGCATGG	
AaPAL-r	ACRTCYTGGTTGTGYTGCTCDGC	
AaPAL-3GSP1	GGAAGCCATGGCAATCTCAG	Gene-specific primers for 5'-RACE-PCR
AaPAL-3GSP2	GGCCGTTGTTGTAGTAGTCG	
AaPAL-5GSP1	GGGTTCTTGATAGGAGCTCG	Gene-specific primers for 3'-RACE-PCR
AaPAL-5GSP2	CATGGTGGCAACTTCCAGG	
AaPAL5GSP3	GATTACGCCAAGCTTCCGCAATGCGCAACCC CTCCTCC	Gene-specific primer for 5'-RACE-PCR
AaPAL1-f	TATCATATGGTGGCCAACAAGCGCGTG	Primers for full-length amplification of AaPAL1 with NdeI restriction sites (underlined)
AaPAL1-r	TTACATATGCTATTGCTCGAACGGGCCCG	
AaPAL2-f	TATCATATGTTGAGCAACAAGCGGGTGAAGTA T	Primers for full-length amplification of AaPAL2 with NdeI restriction sites (underlined)
AaPAL2-r	TTACATATGTCAACCAGTGAAGGGGCCAGG	
qP1-Actin-f	TTTTGAGCAGGAACTGGATAC	Primer sets for quantitative real-time PCR for fragment amplification <200 bp, T_m ranging from 55.9 to 58.2 °C
qP2-Actin-r	ATACCAGCAGCCTCCATAC	
qP1-StP2a-f	GTGTGTGTCCATCTATCACC	
qP2-StP2a-r	AAGACCTCCTCCAACTCC	
qP7-PAL1&2-f	GAAGCTCATGTTTGCCCAG	
qP8-PAL1&2-r	TGGCCAGGAACTGCAGCT	

Suppl. Table S2 Accession numbers of *PAL* nucleotide sequences used for the design of primers for amplification of an internal *PAL* fragment from *Anthoceros agrestis* cDNA

GU456381.1	AY803287.1	AY803283.1	EU071050.1
Y12461.1	AY803281.1	AY803280.1	FN665700.1
L33677.1	AJ002221.1	JQ975419.1	EU650628.1
DQ230992.2	AY803284.1	U16130.1	AY803289.1
NM_001250027.1	JQ277717.1	HM062775.1	AM418586.1
XM_001760443.1	AY803288.1	AY803286.1	

Suppl. Table S3 C_q values for *actin*, *serine threonine protein phosphatase 2a regulatory subunit* (*St-P 2a*) and *PAL* determined by RT-qPCR as used for calculation of relative expression (Pfaffl 2001)

**Actin**															
**Day**	**0**	**1**	**2**	**3**	**4**	**5**	**6**	**7**	**8**	**9**	**10**	**11**	**12**	**13**	**14**
**Replicate 1**	23.29	21.15	20.65	21.44	21.69	22.25	22.28	23.32	23.31	23.49	23.24	23.65	23.61	24.35	24.16
**Replicate 2**	24.39	22.08	21.86	22.75	23.42	23.19	23.29	24.24	24.50	24.46	24.38	24.80	24.79	25.52	25.13
**Replicate 3**	23.66	21.21	20.83	21.60	21.81	22.31	22.43	23.33	23.54	23.51	23.52	23.88	23.88	24.48	24.13
**Replicate 4**	23.57	21.06	20.76	21.39	21.74	22.32	22.35	23.07	23.30	23.37	23.27	23.68	23.62	24.20	24.08
**St-P 2a**															
**Day**	**0**	**1**	**2**	**3**	**4**	**5**	**6**	**7**	**8**	**9**	**10**	**11**	**12**	**13**	**14**
**Replicate 1**	23.95	22.94	22.99	23.33	23.02	23.62	23.26	23.68	24.13	24.04	23.98	24.17	23.83	24.93	24.55
**Replicate 2**	24.71	23.88	23.96	24.37	24.03	24.70	24.32	24.92	25.05	25.02	24.93	25.16	25.07	25.97	25.62
**Replicate 3**	24.17	23.10	23.08	23.58	23.21	24.01	23.45	24.28	24.18	24.15	24.13	24.31	24.18	25.30	24.82
**Replicate 4**	23.67	23.01	22.99	23.31	23.21	23.75	23.28	23.69	24.10	23.89	23.97	24.20	24.10	25.15	24.68
**PAL**															
**Day**	**0**	**1**	**2**	**3**	**4**	**5**	**6**	**7**	**8**	**9**	**10**	**11**	**12**	**13**	**14**
**Replicate 1**	26.42	24.02	23.37	23.66	23.41	24.10	24.29	25.24	26.16	25.56	25.88	26.12	26.76	28.09	28.22
**Replicate 2**	27.34	24.91	24.10	25.08	24.31	25.11	25.07	26.20	27.11	26.39	26.57	26.74	27.48	29.48	28.65
**Replicate 3**	26.59	24.41	23.38	24.18	23.56	24.41	24.51	25.53	26.19	25.92	25.89	26.05	27.34	28.09	28.11
**Replicate 4**	26.34	24.08	23.15	23.76	23.45	24.12	24.08	25.82	26.01	25.51	25.63	25.84	26.77	27.76	28.28

Suppl. Table S4 (Putative) PAL amino acid sequences used for constructing the phylogenetic tree shown in Suppl. Fig. S8

Accession number	Species	Order	Abbreviation
ADO24189	*Allium sativum*	Asparagales	Allsat
MN378319, MN378320	*Anthoceros agrestis*	Anthocerotales	Antagr
NP181241, NP190894, NP196043, NP187645	*Arabidopsis thaliana*	Brassicales	Aratha
ASH96760	*Asarum sieboldii*	Piperales	Asasie
CAH17686	*Beta vulgaris*	Caryophyllales	Betvul
AKN79308	*Betula platyphylla*	Fagales	Betpla
XP_003575240, XP_003575400, XP_003575396	*Brachypodium distachyon*	Poales	Bradis
ASU87402	*Camellia sinensis*	Ericales	Camsin
AKA60049	*Citrus reticulata*	Sapindales	Citret
AEL21617	*Coffea arabica*	Gentianales	Cofara
XP_004143258, XP_004145752, XP_004149916	*Cucumis sativus*	Cucurbitales	Cucsat
AFX98071	*Cunninghamia lanceolata*	Cupressales	Cunlan
BAG31930	*Daucus carota*	Apiales	Daucar
AAW80636	*Diphasiastrum tristachyum*	Lycopodiales	Diptri
AKC03646	*Elaeis guineensis*	Arecales	Elagui
BAG74770, BAG74771, BAG74772, BAG74773	*Ephedra sinica*	Gnetales	Ephsin
AAW80639	*Equisetum arvense*	Equisetales	Equarv
XP_012836017, XP_012834755	*Erythranthe guttata*	Lamiales	Erygut
BAL63070	*Eucalyptus robusta*	Myrtales	Eucrob
ABU49842	*Ginkgo biloba*	Ginkgoales	Ginbil
AFN85669	*Hibiscus cannabinus*	Malvales	Hibcan
AAW80637	*Isoetes lacustris*	Isoetales	Isolac
GAQ82226	*Klebsormidium nitans*	Klebsormidiales	Klenit
AAL55242	*Lactuca sativa*	Asterales	Lacsat
AHA44840	*Larix kaempferi*	Pinales	Larkae
BAM28963, BAM28964	*Lilium hybridum*	Liliales	Lilhyb
BAA24928	*Lithospermum erythrorhizon*	Boraginales	Litery
PTQ29977	*Marchantia polymorpha*	Marchantiales	Marpol
CAA41169	*Medicago sativa*	Fabales	Medsat
CBJ23826	*Melissa officinalis*	Lamiales	Meloff
ACG56647	*Musa acuminata*	Zingiberales	Musacu
XP_010246007, XP_010262867, XP_010253912	*Nelumbo nucifera*	Proteales	Nelnuc
BAA22963	*Nicotiana tabacum*	Solanales	Nictab
XP_024400505, XP_024386700, XP_024393386	*Physcomitrium patens*	Funariales	Phypat
AAA84889, AHX74218	*Pinus taeda*	Pinales	Pintae
AIU99853	*Plagiochasma appendiculatum*	Marchantiales	Plaapp
AAN52280	*Populus tremuloides*	Malpighiales	Poptre

Accession number	Species	Order	Abbreviation
XP_002315308, XP_006381441	*Populus trichocarpa*	Malpighiales	Poptri
AAW80640	*Psilotum nudum*	Psilotales	Psinud
AAF40223	*Rubus idaeus*	Rosales	Rubida
EFJ17024	*Selaginella moellendorffii*	Selaginellales	Selmoe
AAW80643	*Struthiopteris spicant*	Polypodiales	Strspi
CAA68036	*Triticum aestivum*	Poales	Triaes
AEX32784, CBI31445, CBI16336	*Vitis vinifera*	Vitales	Vitvin
XP_008668474, NP_001147433, XP_008645952	*Zea mays*	Poales	Zeamay
KMZ75382	*Zostera marina*	Alismatales	Zosmar

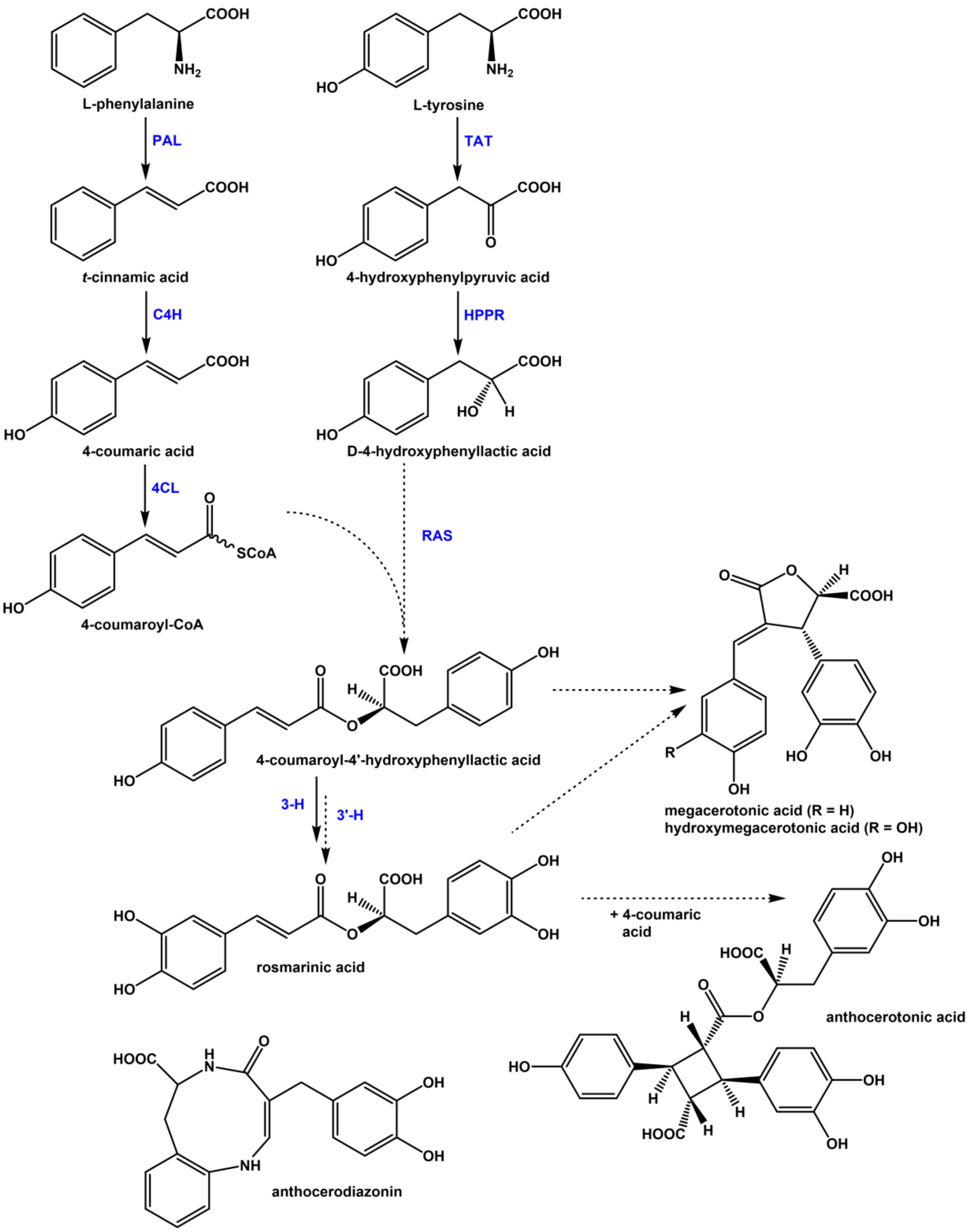

Suppl. Fig. S1 Biosynthetic pathway for rosmarinic acid as elaborated in *Coleus blumei*. Enzymes that have not yet been found and characterized in *Anthoceros agrestis* are shown with dashed arrows. Specialized compounds isolated from *Anthoceros agrestis* that are putatively derived from rosmarinic acid are added. The enzyme involved are: **PAL** – phenylalanine ammonia-lyase, **C4H** – cinnamic acid 4-hydroxylase, **4CL** – 4-coumarate co-enzyme A ligase, **TAT** – tyrosine aminotransferase, **HPPR** – hydroxypyruvic acid reductase, **RAS** – rosmarinic acid synthase, hydroxyphenyllactic acid:hydroxycinnamoyl-CoA hydroxy-cinnamoyltransferase, **3-H**, **3‘-H** – 3-hydroxylase/ 3‘-hydroxylase

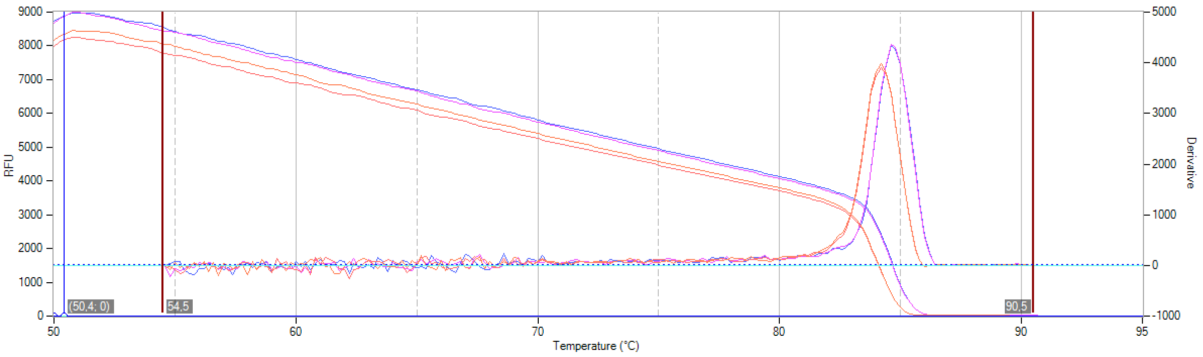

Suppl. Fig. S2 Melting curves for qPCR amplicons of *AaPAL1* (higher T_m) and *AaPAL2* (lower T_m). The two fragments show distinct differences in melting temperature based on their GC content (*PAL1* fragment 59.0%, *PAL2* fragment 56.5%)

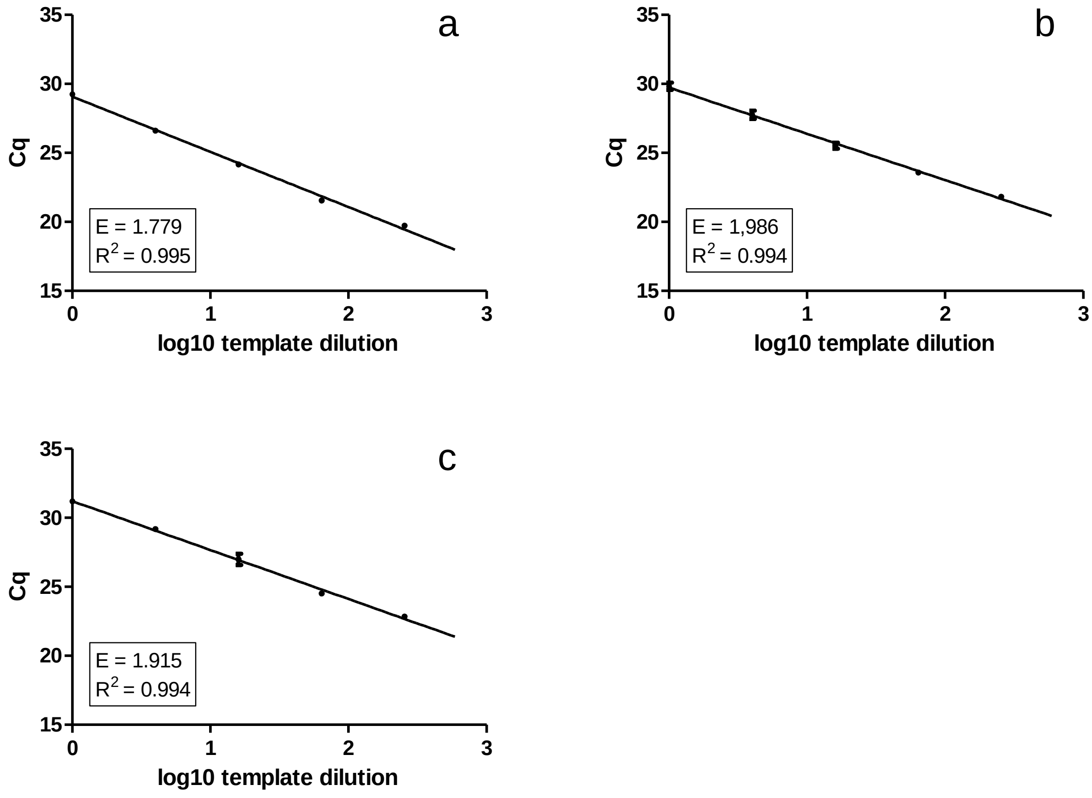

Suppl. Fig. S3 Fourfold dilution series and resulting RT-qPCR efficiencies (E) of *actin* (**a**), *St-P 2a* (**b**) and *PAL* (**c**) (*n* = 3 ± SD)

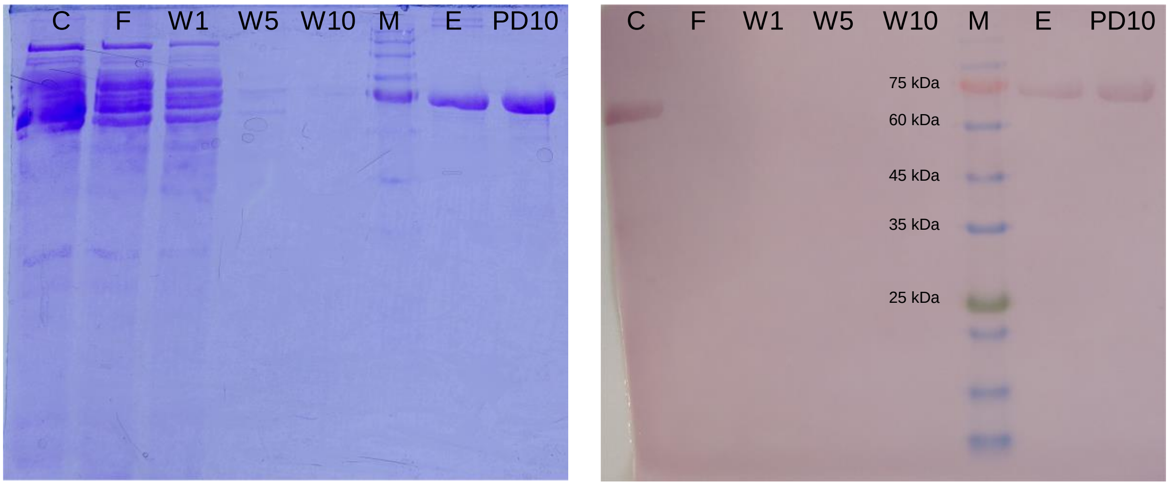

Suppl. Fig. S4 SDS-PAGE (left) and Western blot (right) of a purification over Ni-NTA resin of AaPAL1. Lane C = crude extract, F = flow through, W1, W5, W10 = washing fractions, E = elution fraction, PD10 = elution fraction desalted through PD10 column

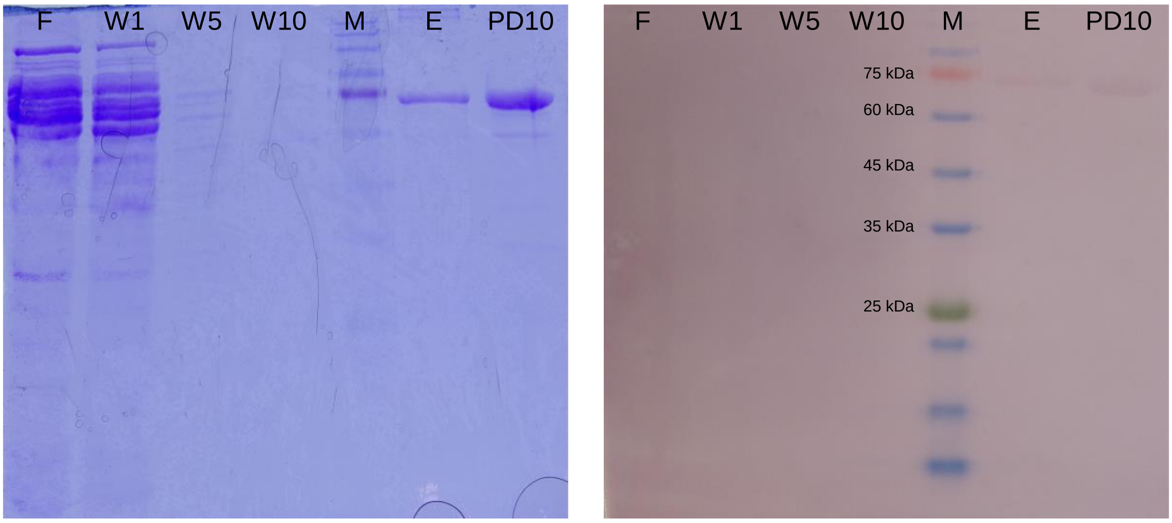

Suppl. Fig. S5 SDS-PAGE (left) and Western blot (right) of a purification over Ni-NTA resin of AaPAL2. Lane F = flow through, W1, W5, W10 = washing fractions, E = elution fraction, PD10 = elution fraction desalted through PD10 column

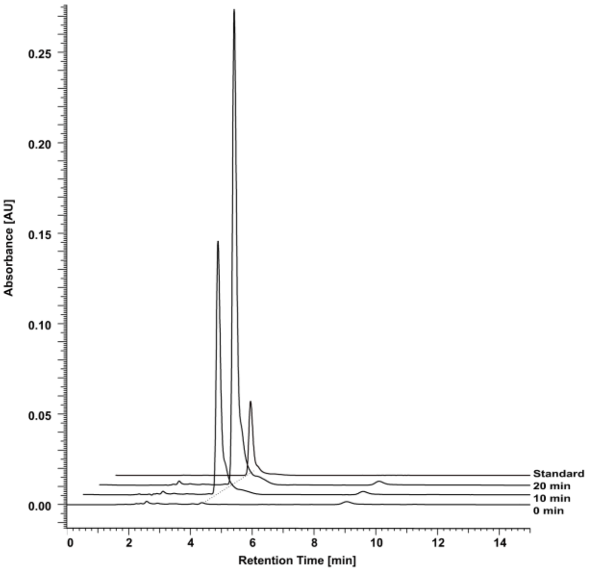

Suppl. Fig. S6 HPLC-chromatograms of PAL assays with L-phenylalanine incubated for 0, 10 and 20 min and *t*-cinnamic acid as standard. HPLC conditions are described in Materials and methods

```
MoPAL       ---------------------------------------MAENGHHDSNGFCVK------    15
AaPAL1      MVANKRVKVVVENGN---GNGALKEDILLQHHENGSGQDHHMETAAVIPKFCFERAGGEI    57
AaPAL2      MLSNKRVKYTGDRGSNGVGVHAAKDELLLGQVVG--ACPMDLDAGARIPNFCFERAGGEI    58
                                                    :       **.:

MoPAL       QNDPLNWVAAAESLKGSHLDEVKRMVEEFRK-PVVKLGGETLTISQVAAIAAKDNAVAVE    74
AaPAL1      VHDPLNWVRTSETLEGSHLDEVKQMVEVFFGSDEVAIEGVNLTVAQVTAIARRADVKVRL    117
AaPAL2      VHDPLNWVRTSEALECSHLDEVKQMVGTFFTADEVAIEGVSLSVAQVTAIARRPGVKVRL    118
             :****** ::*:*: *******:**   *     * : * .*:::**:*** : .. .

MoPAL       LAESARAGVKASSDWVMESMNKGTDSYGVTTGFGATSHRRTKQGGALQKELIRFLNAGIF    134
AaPAL1      DAEAAKQRVDDSSNWVLSNMAKGTDTYGVTTGFGATSHRRTMQGAELQKELIRFLNAGVF    177
AaPAL2      DAEAAKQRVDQSSNWVLANMLKGTDTYGVTTGFGATSHRRTNQGAELQKELIRFLNAGIF    178
             **:*:  *. **:**: .* ****:*************** **. ************:*

MoPAL       GNGTETNHTLPHTATRAAMLVRINTLLQGYSGIRFEILEAITKFLNQNITPCLPLRGTIT    194
AaPAL1      GKGE--GNTLPSTAARAAMLVRTNTLLQGFSGIRWDILCALEKLLNAHVTPKLPLRGTIT    235
AaPAL2      GKSE--GNTLPVAAARAGMLVRTNTLMQGFSGIRWEILEGLEKLLNANITPKLPLRGTIT    236
            *:.   .:*** :*:**.**** ***:**:****::** .: *:** ::** ********

MoPAL       ASGDLVPLSYIAGLLTGRPNSKAVGPNGEPLTAEQAFKLAGVNGGFFELQPKEGLALVNG    254
AaPAL1      ASGDLVPLSYIAGLITARPNSRCFTAEGKEVSAEEGLRIAGVAQP-FVLQPKEGLAIVNG    294
AaPAL2      ASGDLVPLSYIAGVLTGRPNSRAFTCEGKEVTGEEALRLAGVASP-FVLQPKEGLAIVNG    295
            ************::*.****:..  :*: ::.*:.:::***    * ********:***

MoPAL       TAVGSGLASIALFEANILAVLSEVMSAIFAEVMNGKPEFTDHLTHKLKHHPGQIEAAAIM    314
AaPAL1      TAVGAAMASMVCFDANILAVMAEVMSAFFCEAMNGKPEFTDPLTHKLKHHPGQMEAAAIM    354
AaPAL2      TAVGAAMASMVCFDANILAVMAEVMSAFFCEVMNGKPEFTDPLTHKLKHHPGQMEAAAIM    355
            ****:.:**:. *:******::*****:*.*.********* ***********:******

MoPAL       EHILDGSGYVKAAQKLHDMDPLQKPKQDRYALRTSPQWLGPQIEVIRTATKMIEREINSV    374
AaPAL1      EWVLDGSSYMKLAAKLHETDPLKKPKQDRYALRTSPQWLGPQIEVIRSATHSIEREINSV    414
AaPAL2      EWVLDGSSYMKLAAKLHETDPLKKPKQDRYALRTSPQWLGPQVEVIRAATHSIEREINSV    415
            * :****.*:* * *** ***:******************:****:**: ********

MoPAL       NDNPLIDVARSKAIHGGNFQGTPIGVSMDNARLAIASIGKLLFAQFSELVNDFYNNGLPS    434
AaPAL1      NDNPIIDAARSIALHGGNFQGTPIGVSMDNMRLALAAIGKLMFAQFSELVNDYYNGGLPS    474
AaPAL2      NDNPIIDAARSIALHGGNFQGTPIGVSMDNMRLALAAIGKLMFAQFSELVNDYYNNGLPS    475
            ****:**.*** *:**************** ***:*:****:**********:**.****

MoPAL       NLSGGRNPSLDYGFKGSEIAMASYCSELQFLANPVTNHVQSAEQHNQDVNSLGLISSRKT    494
AaPAL1      NLSGGPNPSLDYGFKGAEIAMASYTSELQFLANPVTNHVQSAEQHNQDVNSLGLVSARKT    534
AaPAL2      NLSGGPNPSLDYGFKGAEIAMASYTSELQFLANPVTNHVQSAEQHNQDVNSLGLVSARKT    535
            ***** **********:******* *****************************:*:***

MoPAL       VEALDILKLMSSTYLVGLCQAIDLRHLEENLKHAVKNTVSQVAKRTLTMGANGELHPSRF    554
AaPAL1      AEAVEILKLMTSTYLVALCQAVDLRHLEENMVATVKKTVSLVSKKVLSTGSNGTLLQSRF    594
AaPAL2      AESIEILKLMTSTYLVALCQAVDLRHLEENMVAVVKKVVSSVSKKVLSVDNNGVLLQTRF    595
            .*:::*****:*****.****:********:  .**:.** *:*:.*: . ** *  :**

MoPAL       CEKDLIRVVDREYVFAYIDDPCSATYPLMQKLRQVLVEHALKNGEG-EKNASTSIFQKIE    613
AaPAL1      YEKELLYVVENQPPFTYVDDASSPAYPLMQKLRQVLVEKALKPPKELESDERLTLFQRIP    654
AaPAL2      CEKELLLVVEHQAPFTYIDDAANASYPLMQKLRQVLADRALKNPPAIERDENTSLFLKIS    655
             **:*: **:.:  *:*:**  .*:***********.::***     * :   ::* :*

MoPAL       AFEEELKTLLPKEVESARTALESGNPAIANRIAECRSYPLYKFIREELGADFLTGE-KVV    672
AaPAL1      LFEEELVAALDVDVPAARSAYDKGLAALPNRIQECRTYPLYQFVRSELGTELLCGLIKNG    714
AaPAL2      AFEEELVKLLEEEVTVARTAFDKGAAAVPNRILECRTFPLYNFVRSELGTQILCGFIRNQ    715
             *****   *  :*  **:* :.*  *: *** ***::***:*:*.***:::* *  :

MoPAL       SPGEECDKVFTALSNGLIIDPLLECLQGWNGAPLPIC- 709
AaPAL1      SPGSDFEKVFDAINDGKHIAPLLKVLEGWQGTPGPFEQ 752
AaPAL2      SPGQDIEKVFDAITEGKHVAPLLKVLEGWQGTPGPFTG 753
            ***.: :*** *:.:*  : ***: *:**:*:* *:
```

Suppl. Fig. S7 Alignment of AaPAL1 and AaPAL2 and *Melissa officinalis* PAL (MoPAL, accession no. E1UYU6) amino acid sequences using Clustal Omega. The highest diversity is found at the *N*-terminal end. The catalytic triade forming the MIO group (bold blue letters) is included in the conserved signature sequence of the histidine ammonia-lyase family (blue frames) (Wu et al. 2014)

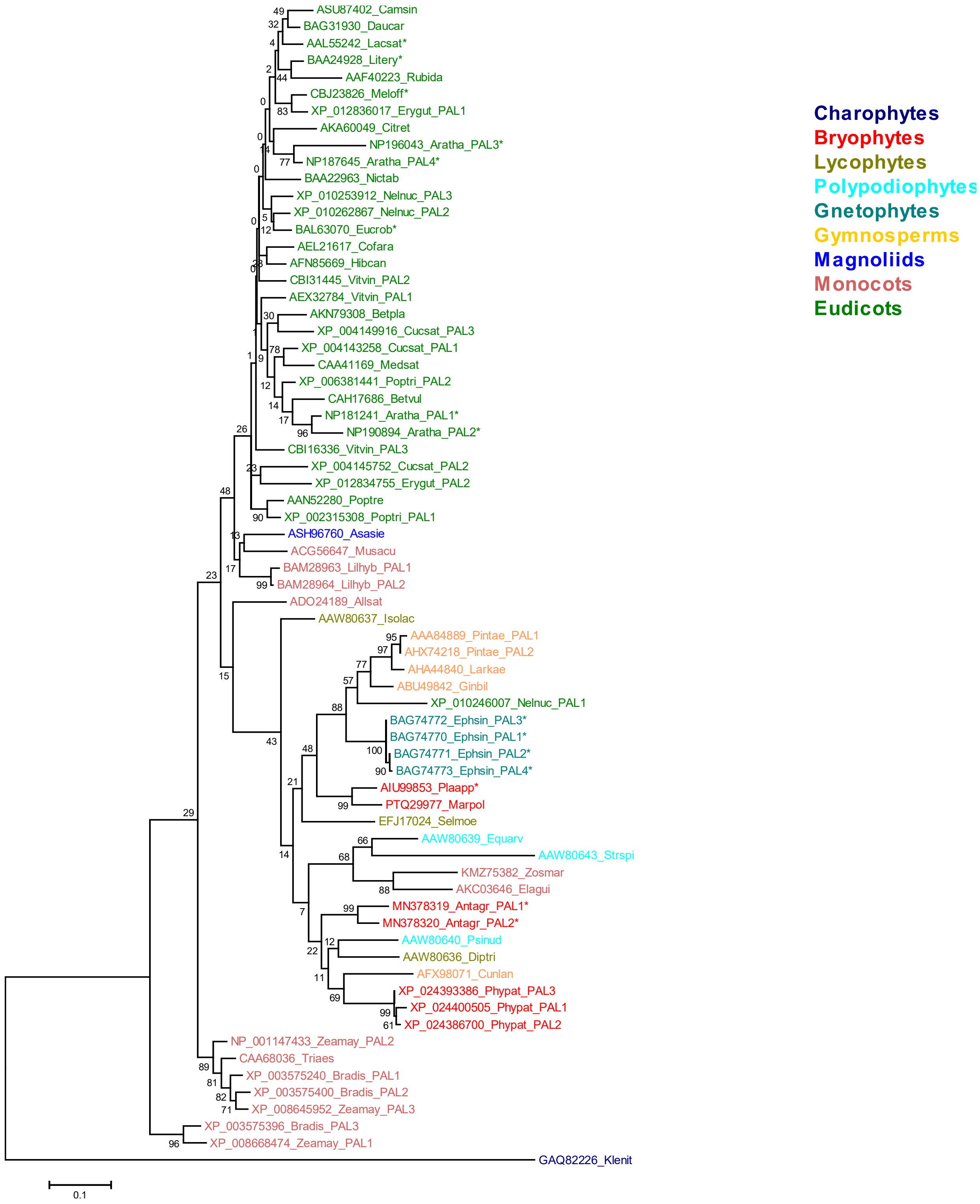

Suppl. Fig. S8 Phylogenetic tree of several PAL amino acid sequences from vascular and non-vascular plants (for accession numbers see Suppl. Table S4) constructed with the MEGA7 software using the maximum likelihood method (Kumar et al. 2016). PAL proteins with proven PAL activity are marked with an asterisk

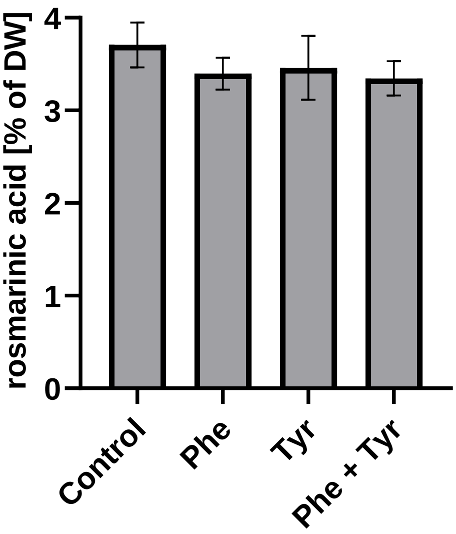

Suppl. Fig. S9 Impact of feeding of 1 mM L-phenylalanine (Phe) or 1 mM L-tyrosine (Tyr) or both to suspension cultures of *Anthoceros agrestis* for 7 days on the content of rosmarinic acid (*n* = 3x2 ± SD)

References for Supplementary material

Kumar S, Stecher G, Tamura K (2016) MEGA7: Molecular Evolutionary Genetics Analysis version 7.0 for bigger datasets. Mol Biol Evol 33:1870-1874. doi.org/10.1093/molbev/msw054

Pfaffl MW (2001) A new mathematical model for relative quantification in real-time RT-PCR. Nucl Acids Res 29:e45. doi: 10.1093/nar/29.9.e45

Wu Z, Gui S, Wang S, Ding Y (2014) Molecular evolution and functional characterisation of an ancient phenylalanine ammonia-lyase gene (NnPAL1) from *Nelumbo nucifera*: novel insight into the evolution of the PAL family in angiosperms. BMC Evol Biol 14:100. doi:10.1186/1471-2148-14-100
